# Difference in structural and chemical properties of sol–gel spin coated Al doped TiO_2_, Y doped TiO_2_ and Gd doped TiO_2_ based on trivalent dopants

**DOI:** 10.1039/c8ra03950j

**Published:** 2018-08-22

**Authors:** Nor Damsyik Mohd Said, Mohd Zainizan Sahdan, Nafarizal Nayan, Hashim Saim, Feri Adriyanto, Anis Suhaili Bakri, Marlia Morsin

**Affiliations:** Microelectronics and Nanotechnology – Shamsuddin Research Centre (MiNT-SRC), Universiti Tun Hussein Onn Malaysia (UTHM) 86400 Batu Pahat Johor Malaysia zainizan@uthm.edu.my; Electrical Engineering Department, Politeknik Kota Kinabalu No. 4, Jln Politeknik, KKIP Barat, Kota Kinabalu Industrial Park 88460 Kota Kinabalu Sabah Malaysia damsyik@polikk.edu.my; Electrical Engineering Department, Sebelas Maret University Jl. Ir Sutami No. 36A-Kentingan Surakarta 57126 Indonesia

## Abstract

In this research, pure titanium dioxide (TiO_2_) and doped TiO_2_ thin film layers were prepared using the spin coating method of titanium(iv) butoxide on a glass substrate from the sol–gel method and annealed at 500 °C. The effects on the structural and chemical properties of these thin films were then investigated. The metal doped TiO_2_ thin film which exists as trivalent electrons consists of aluminium (Al), yttrium (Y) and gadolinium (Gd). The anatase phase of the thin films was observed and it was found that the crystal size became smaller when the concentration of thin film increased. The grain size was found to be 0.487 to 13.925 nm. The types of surface morphologies of the thin films were nanoporous, with a little agglomeration and smaller nanoparticles corresponding to Al doped TiO_2_, Y doped TiO_2_ and Gd doped TiO_2_, respectively. The trivalent doping concentration of the thin films increased with a rising of thickness of the thin film. This can contribute to the defects that give advantages to the thin film when the mobility of the hole carriers is high and the electrons of Ti can move easily. Thus, Ti^3+^ existed as a defect state in the metal doped TiO_2_ thin film based on lattice distortion with a faster growth thin film that encouraged the formation of a higher level of oxygen vacancy defects.

## Abstract 2

Ti^3+^ state in metal doped TiO_2_ based on lattice distortion that encouraged the formation of oxygen vacancy defects.

## Graphical abstract

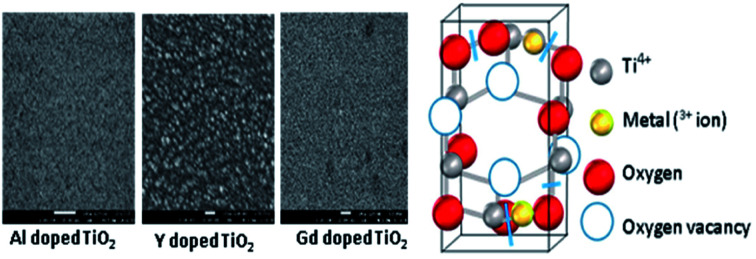

## Introduction

Titanium dioxide (TiO_2_) is nanomaterial with a vast range of applications for energy production,^1^ electronics,^2^ solar devices^3^ and gas sensors at the nanometre length scale. TiO_2_ thin films can be obtained using the sol–gel technique, which is a reliable and low-cost chemical route.^4^ The spin coating method is widely used for the deposition of materials.^5^ TiO_2_ is an n-type semiconductor with low conductivity.^6^ TiO_2_ materials need to be synthesised with a high surface to bulk ratio with a smaller particle size to attain an efficient contact in the target gas and photocatalytic or photochemical reactions arise on the surface of TiO_2_.^7^ Therefore, there has been much effort has been directed to doping TiO_2_ with metal atoms, where the electrons move from one atom to another atom which causes species with an overall electric charge to be formed.^8^ Such species are called ions in which the ionic state is 3+, from the metal atoms. Species with overall positive charges are called cations. Therefore, individual Ti atoms losing electrons give monatomic ions, because of the Ti^4+^ reduction to Ti^3+^.^9^ When Ti atoms lose electrons this causes a change of the characteristic number of electrons in which the holes' carrier mobility of the metal is high and the electrons can move easily. Thus, the ionic state of 3+ charges of metal atoms can also modify TiO_2_, which shows excellent reproducibility.^10^ The Ti^3+^ reactions are one of the effects of point defects. Bharti *et al.* stated that the point defects in terms of charged oxygen vacancies and interstitial titanium ions with three or four charges affect the conductivity of the thin films.^11^ Xiong *et al.* reported that Ti^3+^ plays a role as a defect which increases the conductivity and stability.^9^ Stability here means the orientation phase of the TiO_2_ doped with metal. Yang *et al.* explained that the anatase phase of TiO_2_ thin film also can also contribute to the stability by controlling its parameter properties.^12^ There is also an indication that anatase is the most promising phase because of its higher surface reactivity to gases^13^ and photocatalysts.^14^

Trivalent dopants need to be used to improve the conductivity, decrease grain growth, increase the crystallinity of the peak and increase the surface area. Xu *et al.* showed that the defects which existed in TiO_2_ from a trivalent dopant also contribute excellent results.^15^ Trivalent dopants which can be used for doping are aluminium (Al),^10^ gallium (Ga),^16^ yttrium (Y),^17^ niobium (Nb)^18^ and gadolinium (Gd).^19^ Apart from this, the optimisation of the doping concentration decreases the resistivity of the TiO_2_ and increases the charge carrier concentration.^20^

The aim of this research is to introduce Ti^3+^ as a defect state of the metal doped TiO_2_ thin film based on the lattice distortion in which the oxygen vacancies have been created. Thus, it is first necessary to study the effects of Al, Y and Gd doping on the structural and chemical properties of the TiO_2_ thin film. This study discovered that the differences have been attributed to various causes such as doping elements, phase relations, crystal size, chemical elements, structural defects, surface roughness and grain size of the thin film.

## Experimental

Firstly, for the substrate preparation, a glass sheet with a size of 2.5 cm × 2.5 cm was cut, and this was used as a substrate. The glass was cleaned with acetone in an ultrasonic bath for 5 min at 50 °C. This cleaning process was needed to remove the organic contamination on the glass substrate surface. After that, the substrate was rinsed in deionized water. Then, the substrate was purged to a dry state with nitrogen gas (N_2_). The N_2_ was used to reduce the oxidation, which is established using an inert atmosphere with a very low dew point. The TiO_2_ sol was prepared using a method reported in previous work. The materials used were titanium(iv) butoxide [Ti(OC_4_H_9_)_4_; Sigma-Aldrich, 97%] as a precursor, ethanol (C_2_H_5_OH) as a solvent, deionised water as a source for adding oxygen (O), acids [glacial acetic acid (CH_3_CO_2_H) and hydrochloric acid (HCl)] and Triton X-100 (Sigma-Aldrich) as a stabilizer to avoid precipitation in the solution. Titanium(iv) butoxide mixed with ethanol, acid catalysts, Triton X-100 and aluminium nitrate nonahydrate [Al(NO_3_)_3_·9H_2_O; Sigma-Aldrich, ≥98%] were stirred for 3 h (ageing process) using sol–gel synthesis under ambient conditions to obtain the gel at room temperature. Dopant precursors such as aluminium nitrate nonahydrate, yttrium(iii) nitrate hexahydrate (N_3_O_9_Y·6H_2_O; Sigma-Aldrich, 99.8%) and gadolinium(iii) acetate hydrate (C_6_H_9_GdO_6_·*x*H_2_O; Sigma-Aldrich, 99.9%) were added last. All the steps for the undoped sample and doping samples were executed sequentially. A metal ion doped TiO_2_ solution can be used for the deposition of thin films using the spin-coating method. The acquired sol was spin-coated on the glass substrate at a speed of 3000 rpm over 30 s to deposit five layers of uniform films. The TiO_2_ solution was dropped up to 10 times onto the substrates. After spin-coating, the layers formed were preheated at 100 °C for 5 min. All the layers were annealed at 500 °C for 1 h to achieve better crystallization. The annealing process was a heat treatment process which altered the microstructure of the material to change its mechanical or electrical properties. After the annealing process, the thin films needed to be cooled in ambient air to room temperature. Finally, the TiO_2_ thin films were structurally and chemically, characterised.^21^ The structural properties were characterized using a PAnalytical Smartpowder X-ray diffractometer (XRD). XRD is a non-destructive technique which can be used to detect the crystalline phases of unknown material phases by comparing them with records of the crystal structures of materials in the Inorganic Crystal Structure Database (ICSD). The surface morphologies and cross-sections of the thin films were observed using field emission scanning electron microscopy (FESEM). The surface morphology images were magnified by 200 K and 100 K. The magnification of the thickness images was 100 K. The surface topologies and roughness were characterized using a Park Systems XE-100 atomic force microscope (AFM), at room temperature. In this research, a non-contact mode was used in which the cantilever oscillates almost on the surface of the sample and senses the van der Waals attractive force that causes a frequency move in the resonant frequency of a stiff cantilever. Furthermore, it was also realised that it was necessary to use the information on grain size and roughness of the TiO_2_ thin film. X-ray Photoelectron Spectroscopy (XPS) surface chemical analysis techniques were used to determine a stoichiometry change of the oxide for the uppermost top layer of the chemical materials' surface.

## Results and discussion

**Fig. 1:**
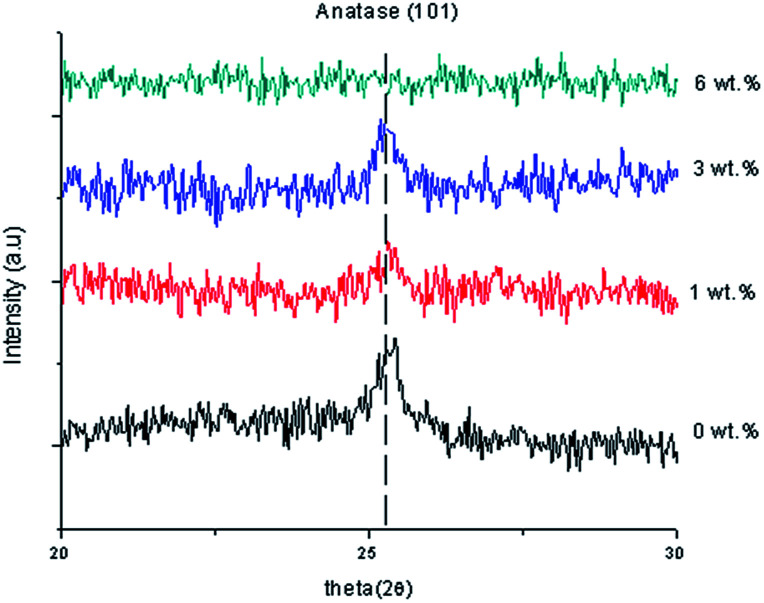
XRD data of the undoped thin film and Al doped TiO_2_ thin film.

Fig. 1 shows the XRD data of the TiO_2_ thin films, as-deposited undoped thin film and Al doped TiO_2_ thin film at different doping concentrations. The anatase TiO_2_ peaks (*I*4_1_/*amd*) became smaller with an increase of the Al doping concentration because of the disorder caused by the size of ionic radii of Al^3+^ and Ti^4+^. Doping of Al into TiO_2_ can lead to a crystallite sized anatase TiO_2_. Thus, this indicates that the crystallite sizes were smaller when compared to the undoped thin films.

Al doped TiO_2_ has a smaller average size than the pure TiO_2_. This phenomenon can be described as the quantum size effect, because the accumulation of Al causes a significant decrease in the crystal size as shown Table 1. The formula of crystal size (*D*) is simplified as:1
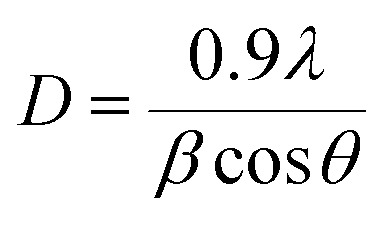


**Table 1:** Peak properties of Al doped TiO_2_ thin films at different doping concentrations

Al doped TiO_2_ con.	Position, 2 theta (°)	Intensity (cts)	FWHM (°)	Calculated crystallite size (*D*, nm)	Dislocation (×10^15^)	Strain	Stress (GPa)	Lattice constants	
								*a*	*c*
0 wt%	25.3735	233.83	0.36	22.62	1.954	0.147	0.34	3.7760	9.4860
1 wt%	25.3574	108.8	0.3542	22.62	1.892	0.105	−0.245	3.7800	9.5100
3 wt%	25.2528	103.7	0.432	18.85	2.814	0.589	1.37	3.7960	9.4440

Strain (*ε*) is defined as the fractional change in length and the dislocation density (*δ*) is defined as the length of dislocation lines per unit volume of the crystal, and it is measured from the following relationship using the simple approach of Freund and Suresh^22^ (see eqn (2)).2
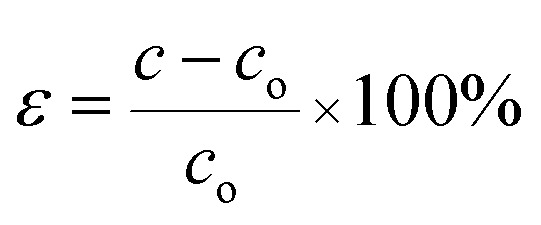
3
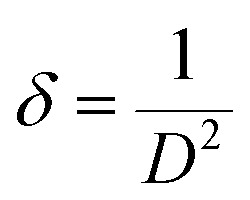


**Fig. 2:**
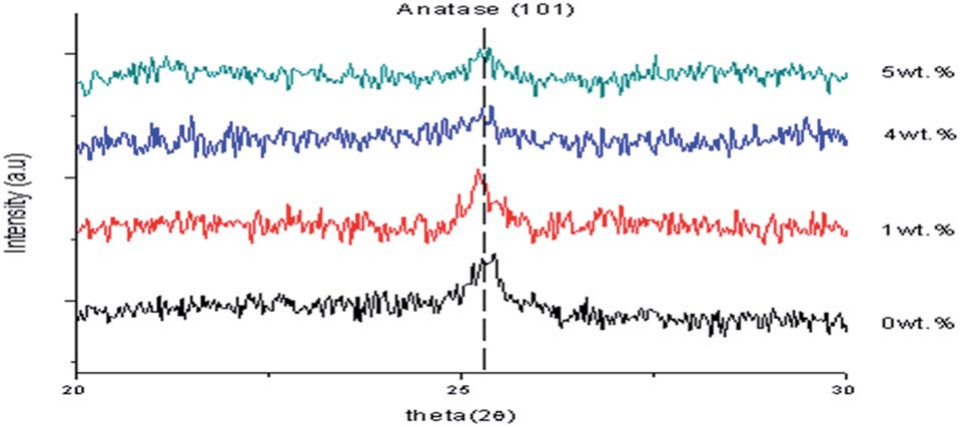
XRD data of the undoped thin film and Y doped TiO_2_ thin film.

The strain and dislocation density increases with the increase in doping concentration and was attributed to the combination of the atoms in TiO_2_ in the substitution. The stress (*σ*) of the prepared thin films is calculated using:^22^4
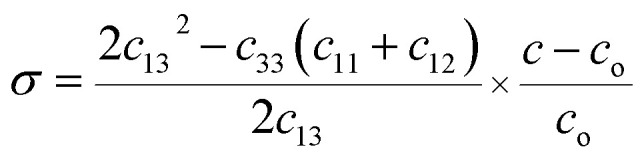
where, *c*_11_ = 208.8 GPa, *c*_33_ = 213.8 GPa, *c*_12_ = 119.7 GPa and *c*_13_ = 104.2 GPa.^23^ Doping with a larger amount of dopant with 6 wt% of Al caused no characteristic peaks to be observed and the peaks related to Al were not detected, suggesting that Al did not form a significant second phase. This means that the XRD did not detect the dopant phase either, because of the low concentration of Al doping. The samples became amorphous when a higher Al doping concentration was applied. This also applied to the optimisation of Al substitution on the Ti sites. When an atom with a larger atomic radius replaced a small atom in the lattice, it was observed that the peak was shifted towards the lower 2*θ* value. The crystallinity of the film influences the transport properties of the photo-generated electrons and holes to the film surface as well as the bandgap energy.^24^ A lattice is defined as the periodic arrangement of atoms in the crystal. The smaller the ions or the higher the charge on the ions, the stronger is the attraction between the ions. This creates a strong bond. Lattice energy is the measurement of the strength of the ionic bond. The stronger the ionic bond, the more exothermic is the lattice energy. The lattice energy relies on the size of the ions and the charge on the ions^25^ Therefore, during the formation of ionic bonds, the ionization energy required should be low and at the same time the electron affinity should be high, meaning that the reaction is more exothermic. The anatase peak of pure TiO_2_ thin film was measured at 25.3735° which corresponded to ICSD file no. 98-002-4276. The anatase peak with 1 wt% Al doping concentration was measured at 25.3574° which corresponded to ICSD file no. 98-020-0392 and the anatase nanocrystalline peak with 3 wt% Al doping concentration was measured at 25.2528° which corresponded to ICSD file no. 98-015-4601. Table 1 shows the lattice constants and crystallite size of the synthesised samples. The position of 2*θ* was shifted to the left and the intensity decreased when the Al doping concentration increased because of the increase in the arrangement of the atoms. In addition, the full width at half maximum (FWHM) became wider and the crystal size decreased because of the increase of the dislocation of the Al doping concentration. When 1 wt% Al doping concentration was applied, the strain of the thin film decreased, so the stress caused a compressive thin film because of the negative sign. However, when 3 wt% Al doping concentration was applied, the strain of the thin film increased, so the stress of the thin film caused a tensile thin film because of the positive sign. Pure TiO_2_ has a lattice constant of *a* = 3.7760 and *c* = 9.4860. The lattice of *a* and *c* were changed to *a* = 3.7960 and *c* = 9.4440 when Al doping concentration 3 wt% was applied. Doping Al atoms increased the lattice constant “*a*” of the tetragonal TiO_2_ structure which may show that Al has entered into the TiO_2_ lattice. However, when a 3 wt% Al doping concentration was applied, the crystallite size was 18.85 nm. A comparison of the (101) peaks of as-synthesized undoped and Al doped TiO_2_ showed that the FWHM increased based on doping concentrations. Furthermore, the trend was valid for all peaks of the samples as well. Fig. 2 reveals the XRD patterns of the TiO_2_ nanoparticles, which show the apparent characteristic peaks of the anatase phase. The anatase nanocrystalline peak of 1 wt% Y doping concentration was measured at 25.2599° which corresponded to ICSD file no. 98-015-4601. The anatase peak of 4 wt% Y doping concentration was measured at 25.2065° which corresponded to ICSD file no. 98-000-9854 and the anatase nanocrystalline peak of 5 wt% Y doping concentration was measured at 25.2887° which corresponded to ICSD file no. 98-015-4602. No other phases were detected and at a larger than 5 wt% Y doping concentration indicated that they were amorphous in nature. Likewise, the intensity of the diffraction peaks decreased with increasing Y doping concentration in the TiO_2_ lattice. This can be ascribed to Y impurities dislocating the crystal structure of the anatase TiO_2_. This could be because some Ti^4+^ was replaced by Y^3+^ and by some Y^3+^ entering into the TiO_2_ lattice, or the yttrium(iii) oxide (Y_2_O_3_) content was an extremely small size and could not be detected. XRD patterns of pure and Y doped TiO_2_ nanoparticles show that the characteristic peaks appear with wider FWHM and the intensity of the diffraction peaks decreased with an increase in the Y doping concentration. The ionic radius of Y^3+^ was 0.088 nm, which was much larger than that of Ti^4+^ at 0.074 nm, so as Y^3+^ entered into the lattice of TiO_2_, there was a lower diffraction intensity, and a wider diffraction peak and the crystal lattice changed, thus resulting in lower crystallinity. These results show that the appropriate Y doping concentration can maintain the crystallinity of TiO_2_ nanoparticles. From the XRD analysis results, it was found that there were differences in the lattice constants between Y doped and pure TiO_2_ thin film, which also inferred that Y^3+^ entered into the lattice of TiO_2_ and substitutes for the Ti^4+^ ion which would eventually be beneficial for separating the charge carriers, extending their lifetime and effectively hindering the recombination of the electron–hole pairs.^17^

The position of 2*θ* was shifted to the left and the intensity decreased when the Y doping concentration increased because of the increase in the arrangement of the atoms. Furthermore, the FWHM became wider and the crystal size decreased because of the increase in the dislocation of the Al doping concentration. When a 1 wt% Y doping concentration was applied, the strain of the thin film increased, so the stress caused it to become a tensile thin film because of the positive sign. However, when a 4 wt% Y doping concentration was applied, the strain of the thin film increased, so the stress caused it to become a compressive thin film because of the negative sign.

The lattice constants of *a* and *c* were altered to *a* = 3.7970 and *c* = 9.5790 when 4 wt% Y doping concentration was applied as shown in Table 2. Doping Y atoms increased the lattice constant “*a*” of the tetragonal TiO_2_ structure, which may show that Y has entered into the TiO_2_ lattice. This shows that an increase in doping concentration weakens the film's crystallinity, which may be because of the stresses caused by the difference in ion size between titanium and the dopant and the segregation of dopants in the grain boundaries for high doping concentrations. The calculated crystallite sizes of Y doped TiO_2_ thin films with 1 wt%, 4 wt% and 5 wt% were 17.2 nm to 45.23 nm. A comparison of the (101) peaks between the undoped and Y doped TiO_2_ shows that the FWHM increased based on the doping concentrations.^26^

**Table 2:** Peak properties of Y doped TiO_2_ thin films at different doping concentrations

Y doped TiO_2_ con.	Position, 2 theta (°)	Intensity (cts)	FWHM (°)	Calculated crystallite size, (*D*, nm)	Dislocation (×10^15^)	Strain	Stress (GPa)	Lattice constants	
								*a*	*c*
1 wt%	25.2599	225.56	0.18	45.23	0.488	0.589	1.37	3.7960	9.4440
4 wt%	25.2065	105.65	0.432	18.84	2.817	0.832	−1.94	3.7970	9.5790
5 wt%	25.2887	131.65	0.4723	17.21	3.376	0.947	−0.221	3.7990	9.5090

**Fig. 3:**
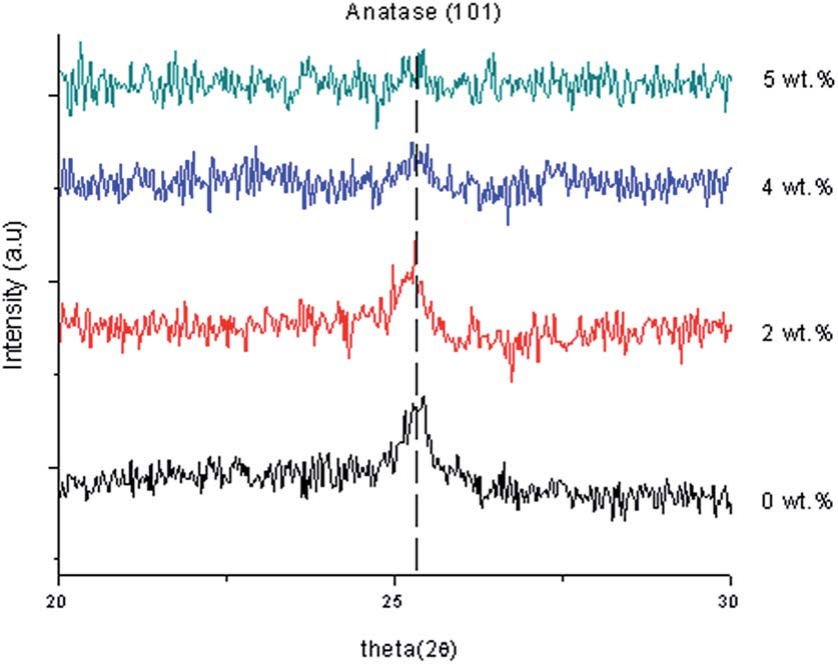
XRD data of the undoped thin film and Gd doped TiO_2_ thin film.

Fig. 3 shows XRD patterns of undoped and Gd doped TiO_2_ thin film using 2 wt%, 4 wt% and 5 wt% of Gd. The TiO_2_ indicated a dominant anatase phase for Gd-undoped sample. Then, no secondary phase was detected for Gd doped and undoped TiO_2_ thin films and more than 4 wt% Gd doping concentration indicated that they were amorphous in nature. The Gd dopant did not cause any shift in peak position of TiO_2_ which may be because the amount of Gd doping was too small. Gd doped TiO_2_ has smaller average size particles than the pure TiO_2_ as shown in Table 1. The diffraction peak of anatase indicated an offset to the lower angle region after doping Gd ions into the anatase TiO_2_ lattice and this demonstrated the incorporation of Gd^3+^ ions into TiO_2_ lattice by substituting them for the Ti^4+^ ion. The Gd^3+^ (with an ionic radius of 0.0938 nm) doping into Ti sites was challenging because of the large difference in the ionic radii. The results of the XRD studies left a lot of uncertainty about the content of crystalline and amorphous phases in the deposited thin films, and only verified that the modification of the chemical composition of deposited Gd doped TiO_2_ thin film really affects the growth mechanism, which has a direct impact on the microstructure.^27^

The anatase nanocrystalline peak of 2 wt% Gd doping concentration was measured at 25.2608° which corresponded to ICSD file no. 98-015-4601. The anatase peak of 4 wt% Gd doping concentration was measured at 25.3202° which corresponded to ICSD file no. 98-008-2084 and the anatase nanocrystalline peak of 5 wt% Gd doping concentration was measured at 25.2887° which corresponded to ICSD file no. 98-015-4601. Table 3 shows the lattice constants and crystallite size of the synthesized samples. The position of 2*θ* was shifted to the left and the intensity of the thin film decreased when the Gd doping concentration increased because of the increase in the arrangement the atoms. Furthermore, the FWHM became wider and the crystal size decreased because of the increase of the dislocation of the Gd doping concentration. When 1 wt% Gd doping concentration was applied, the strain of the thin film increased, so the stress created a tensile thin film because of the positive sign. When 4 wt% Gd doping concentration was applied, the strain of the thin film decreased, so the stressed thin film was still a tensile thin film. With 5 wt% Gd doping concentration, the strain value increased and the stressed thin film still remained as a tensile thin film. The lattice constants of *a* and *c* were changed to *a* = 3.7930 and *c* = 9.5090 when 4% Gd doping concentration was applied. However, doping with Gd atoms increased the lattice constant “*a*” of the tetragonal TiO_2_ structure, which may indicate that Gd had entered the TiO_2_ lattice. This suggested that an enlargement in the doping concentration reduces the crystallinity of the film and that this maybe because of stresses effected by the difference in ion radius size between titanium and the dopant, and the segregation of dopants in the grain boundaries for high doping concentrations. The calculated crystallite sizes of Gd doped TiO_2_ with 2 wt% and 4 wt% corresponded to 18.85 nm and 14.14 nm, respectively. Broadening of the crystallite was present and tended to decrease as a function of 2*θ*. A comparison of the (101) peaks between undoped and Gd doped TiO_2_ showed that the FWHM increased based on the doping concentrations. ^28^

**Table 3:** Peak properties of Gd doped TiO_2_ thin films at different doping concentrations

Gd doped TiO_2_ con.	Position, 2 theta (°)	Intensity (cts)	FWHM, (°)	Calculated crystallite size, (*D*, nm)	Dislocation (×10^15^)	Strain	Stress (GPa)	Lattice constants	
								*a*	*c*
2 wt%	25.2608	206.12	0.432	18.85	2.814	0.589	1.37	3.790	9.4440
4 wt%	25.3202	96.9	0.576	14.14	5.002	0.032	0.07	3.7830	9.4970
5 wt%	25.256	47.02	0.432	18.85	2.814	0.589	1.37	3.7960	9.4440

**Fig. 4:**
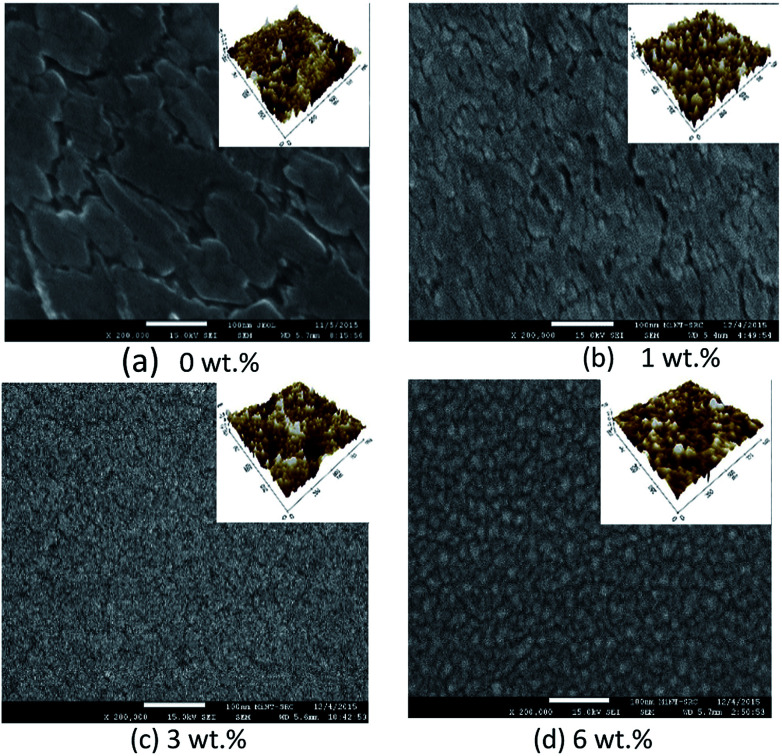
Surface morphology of FESEM micrographs of pure and Al doped TiO_2_ thin films.

**Fig. 5:**
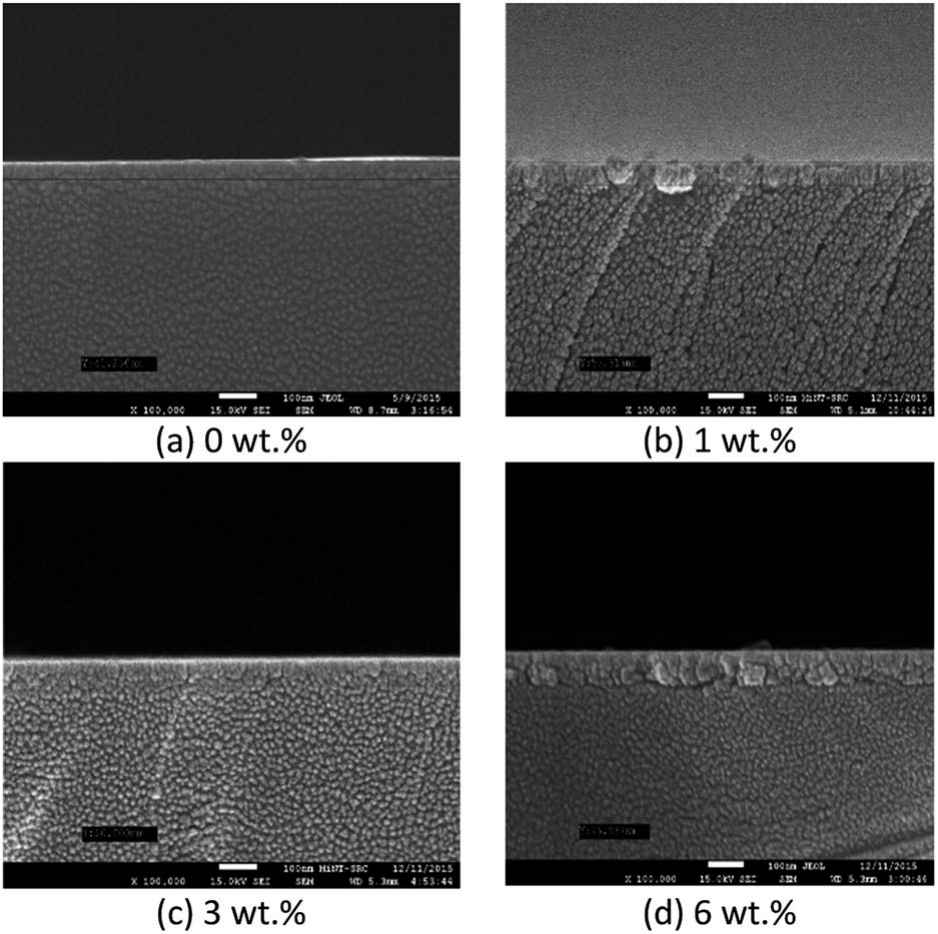
Cross-sectional view of FESEM micrographs of pure and Al doped TiO_2_ thin films.

Fig. 4 and 5 show the surface morphology and cross-sectional area of an FESEM micrograph of pure and Al doped TiO_2_ thin films prepared using the sol–gel method. The Al doping concentrations, 1 wt%, 3 wt% and 6 wt% and their thicknesses are shown in Table 4. This shows the thickness, and verified the increase when the concentration of the Al doping increased. Al doped TiO_2_ thin films revealed the size of nanoparticles to be between 14 nm and 49 nm. The nanoparticle sizes decreased when the Al doping concentration process decreased, resulting in the nanoporous porosity. In order to determine the roughness of the surface as a function of the Al concentration, it was necessary to perform AFM. Fig. 4 depicts the two-dimensional AFM images of the deposited films using 1 wt%, 3 wt% and 6 wt% concentrations of Al doping. Table 4 shows that the grain size increased and the surface roughness of the Al–TiO_2_ thin film decreased. The lower roughness value showed that there was good homogeneity of the Al–TiO_2_ nanoparticles on the surface. The change in the grain shape was determined by analysing the XRD data. Based on the XRD data it can be seen that there is considerable variation in the maximum intensity peak, indicating the preferred growth direction of TiO_2_ for each phase formed with a decreasing Al doping concentration. The decomposition reaction kinetics at the substrate surface defined the grain shape and size. Larger grain sizes provided higher surface contact between the Al doped TiO_2_ thin films and the electrode thus improving electron migration.^29^

**Table 4:** Thickness, grain size and roughness with different Al doping concentration

Al doping concentration	Thickness (nm)	Grain size (nm)	Roughness (nm)
0 wt%	41.250	44.084	3.914
1 wt%	55.313	69.000	0.792
3 wt%	60.000	68.004	0.863
6 wt%	75.937	103.00	0.487

**Fig. 6:**
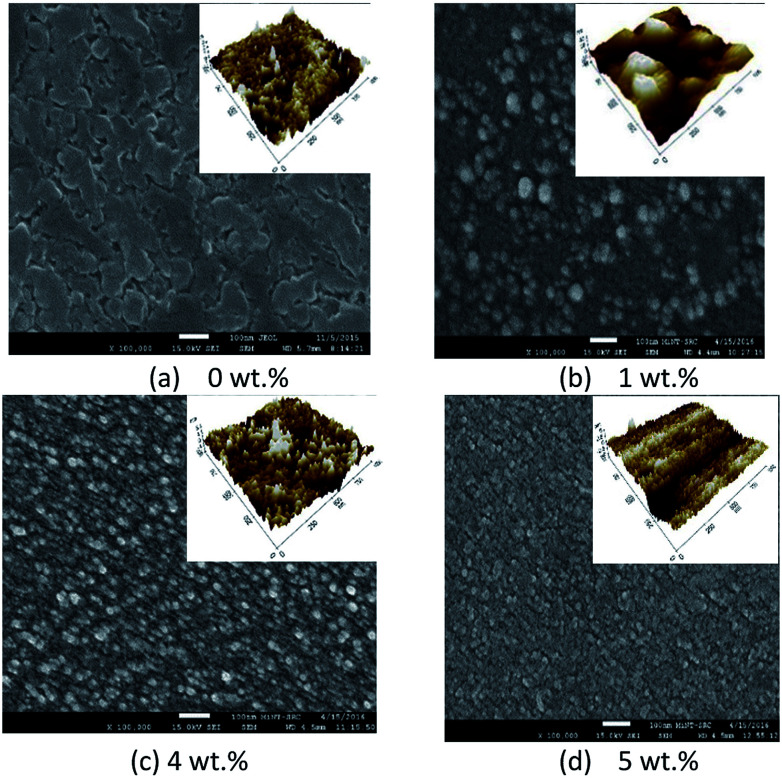
Surface morphology of FESEM micrographs of pure and Y doped TiO_2_ thin films.

**Fig. 7:**
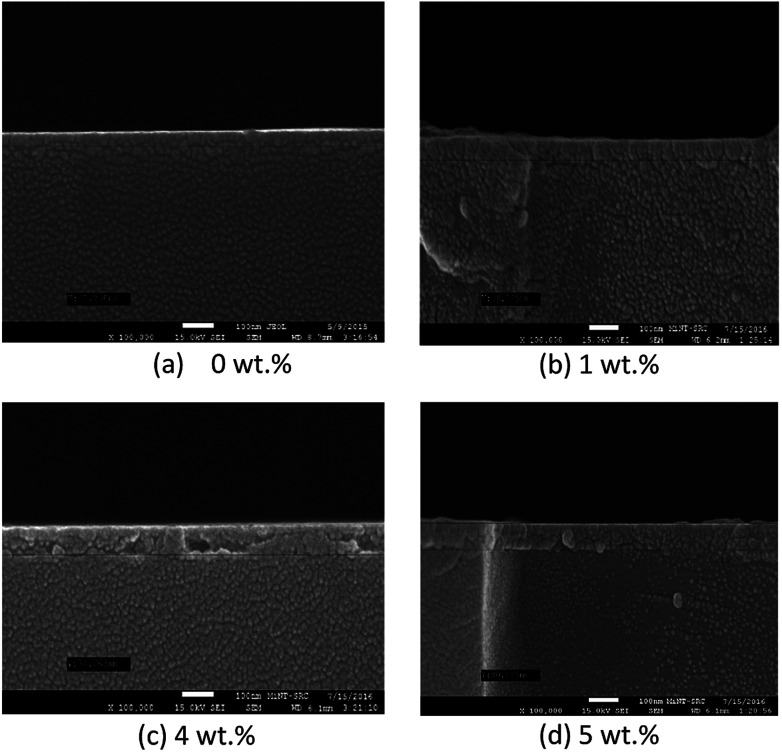
Cross-sectional view of FESEM micrographs of pure and Y doped TiO_2_ thin films.

Fig. 6 and 7 show the surface morphology and the cross-sectional view of Y doped TiO_2_ thin films for all thin film samples. For samples deposited using a Y doping concentration, the thicknesses are shown in Table 5. This shows the increasing thickness that occur when the concentration of the Y doping increased. Morphological properties of the undoped and Y doped TiO_2_ thin films were examined using FESEM, and the images of the grainy looking structure became less grainy with Y doped in the TiO_2_ at 1 wt%, 4 wt% and 5 wt% concentrations. Nevertheless, the granules became smaller at 4 wt% doping concentration and the morphology shifted to a more uniform film structure. The AFM images of pure and Y doped TiO_2_ were prepared by using the spin coating method with yttrium(iii) nitrate [Y(NO_3_)_3_] as a doping source as shown in Fig. 7. The Y doping had little influence on the morphology of the TiO_2_ thin film when the Y doping concentration was increased. It can be seen that the crystalline size of the nanoparticles was about 20 nm to 49 nm. The crystalline size of pure TiO_2_ nanoparticles was about 45.23 nm with a little agglomeration. With the increase in Y doping content, the crystalline size increased, and the agglomeration gradually increased when the Y doping content was at 1 wt%. The crystalline size of the nanoparticles increased to about 49 nm, and the agglomeration became more serious. This was mainly because of the sol–gel process, where the precursor of titanate reacted with NO_3_^−^ and Ti(NO_3_)_2_ was obtained when the doped source Y(NO_3_)_3_ was added. Therefore, NO_3_^−^ can also act as a chelating agent as well as a doping agent, both of which can slow down the condensation reaction rate, thus controlling the nucleation and growth process. Instead, the Y elements can strongly prevent the conversion of the TiO_2_ crystal phase. The AFM roughness analysis also shows the values of the roughness parameters.^30^

**Table 5:** Thickness, grain size and roughness with different concentrations of Y doping

Y doping con.	Thickness (nm)	Grain size (nm)	Roughness (nm)
0 wt%	41.250	44.084	3.914
1 wt%	58.125	40.760	13.925
4 wt%	74.063	44.927	2.040
5 wt%	80.625	63.000	0.979

**Fig. 8:**
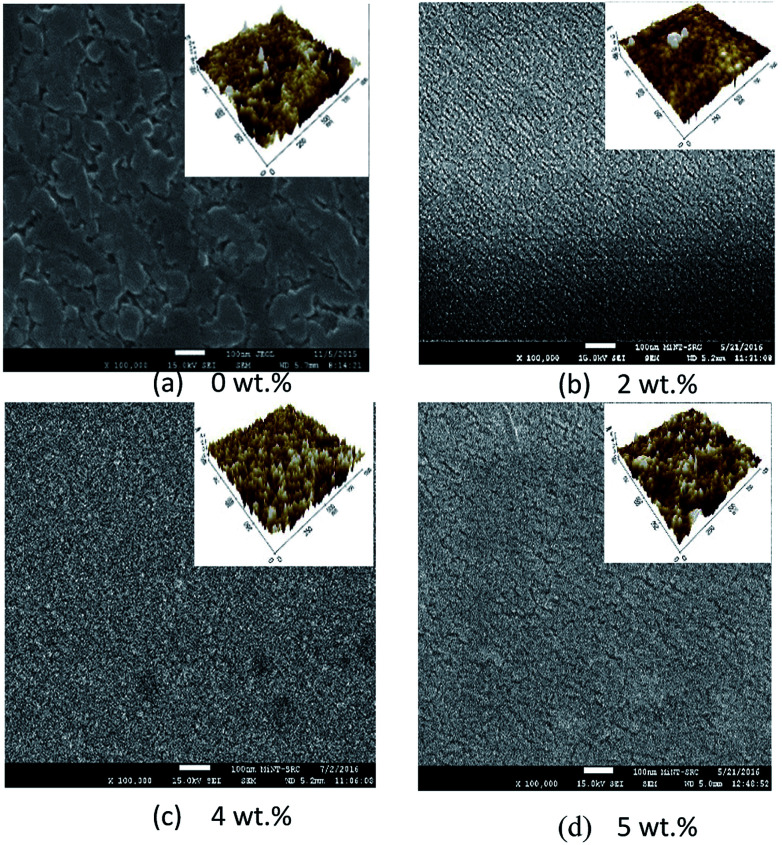
FESEM micrographs showing the surface morphology of pure and Gd doped TiO_2_ thin films.

**Fig. 9:**
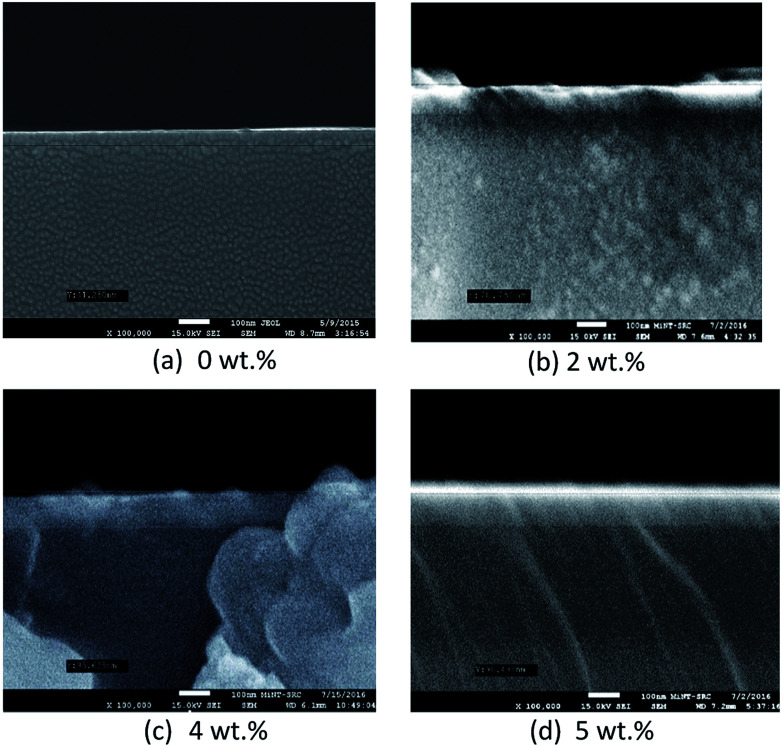
FESEM micrographs showing a cross-sectional view of pure and Gd doped TiO_2_ thin films.

Fig. 8 and 9 show the surface morphology and cross-sectional view of Gd doped TiO_2_ thin films. The thicknesses are shown in Table 6. This shows the increasing thickness when the concentration of Gd doping was increased. Fig. 9 shows that the surface morphology of the Gd doping samples were nearly nanoparticles and had high porosity as the pure TiO_2_ and Gd doped nanoparticles became very small when an increase in the doping process was observed. Fig. 9 also reveals that the two-dimensional size was increased and as well there was an increase in roughness as shown in Table 6. The surface roughness determined the number of active surface sites. In addition, the lower roughness value indicated that the TiO_2_ particles had a very smooth surface. The AFM image shows that at 5 wt% Gd doping the thin film had the lowest roughness. The average grain sizes range around 40.760 nm to 63.000 nm. The changes in the grain shape were explained by examining the XRD data.^28^ When the AFM images of the deposited films were examined, it was found that as the Gd doping increased, then the grain size also increased and the roughness decreased. The thickness increased with increasing Gd doping concentration. It can be clearly observed from the results in Table 6 that the thickness is directly proportional to the Gd doping concentration.

**Table 6:** Thickness, grain size and roughness with different Gd doping concentrations

Gd doping con.	Thickness (nm)	Grain size (nm)	Roughness (nm)
0 wt%	41.250	44.084	3.914
2 wt%	78.750	47.157	1.637
4 wt%	95.625	63.190	0.759
5 wt%	98.438	66.000	0.588

**Fig. 10:**
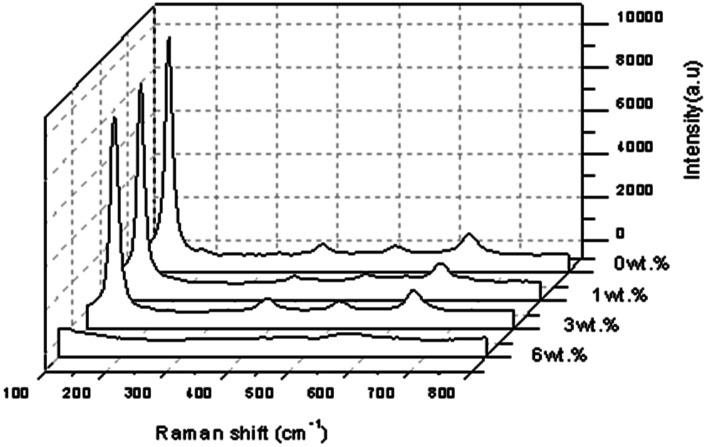
Raman spectroscopy scans for Al doped TiO_2_ thin film.

The Raman spectrum of the pure TiO_2_ at a spectral range of 100–800 cm^−1^ showed that four of the peaks were prominent and the corresponding modes at 144.913 cm^−1^ (E_g_), 398.918 cm^−1^ (B_1g_), 517.139 cm^−1^ (a combination of A_1g_ and B_1g_) and 636.698 cm^−1^ (E_g_), were assigned to the anatase phase as shown in Fig. 10. The spectra of the doped TiO_2_ were slightly shifted as a result of the crystal structure modification from the doping. Raman spectroscopy was carried out to confirm the phase identification of the Al doped TiO_2_ thin films as well as to investigate the defects in the materials as shown in Fig. 9(a). The E_g_ mode of the pure TiO_2_ at 144.913 cm^−1^ arose from external vibrations of the anatase structure and indicated the formation of a long-range order, thus verifying the XRD data. Fig. 10 shows the room temperature Raman spectra of the Al doped TiO_2_, its E_g_ mode when 1 wt% of Al is used remains at 144.913 cm^−1^ but when a concentration of 3 wt% of Al is applied then a high intensity of Raman peak at 145.723 cm^−1^ was observed.

The sharpest and strongest peak at about 145.723 cm^−1^ was assigned to the high frequency branch of the E_g_ mode of Al doped TiO_2_, which was the strongest mode in the anatase phase. The strong E_g_ mode indicated good crystallinity. Thus, from the Raman studies it was determined that the anatase phase was not altered by the presence of a trivalent aluminium dopant. It was noted that doping was considered to be the main factor that would cause the lattice distortion of the crystals, and this is usually due to the differences of the ionic radii of different elements. In this study, the crystallite quality of the Al doped TiO_2_ thin film with 3 wt% of Al was better than the crystallite quality of the other sample of TiO_2_ with a concentration of 1 wt% of Al doping.^31^ The crystallite quality of the doped TiO_2_ with 6% of Al was worse than with the other concentrations because no peak for the anatase phase of TiO_2_ was detected. Furthermore, FWHM showed pure TiO_2_ > Al doped TiO_2_ at the strongest peak, meaning that the FWHM decreased when the doping concentration increased.

**Fig. 11:**
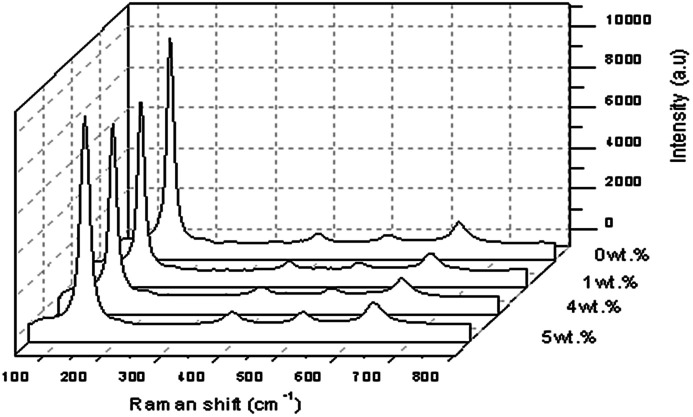
Raman spectroscopy spectra for Y doped TiO_2_ thin film.

Fig. 11 shows the Raman spectra of TiO_2_ with 1 wt%, 4 wt% and 5 wt% of Y concentration at a spectral range with high intensity Raman peaks of anatase TiO_2_ and no Raman peak for the rutile phase was noted. Four normal modes were observed at 144.913 cm^−1^ (E_g_), 395.864 cm^−1^ (B_1g_), 517.139 cm^−1^ (a combination of A_1g_ and B_1g_) and 636.698 cm^−1^ (E_g_). The sharpest and strongest peak at about 144.913 cm^−1^ was assigned to the high frequency branch of the E_g_ modes of TiO_2_, which was the strongest mode in the anatase phase. The strongest E_g_ mode indicated good crystallinity as the Y doping concentration reached a concentration of 5 wt% of Y. Thus from the Raman studies it could be determined that the anatase phase was not changed by the presence of the trivalent yttrium dopant. It can also be observed that doping was considered to be the main factor that would cause the lattice distortion of the crystals, and this verified the XRD data, for it was generally different from the ionic radii of different elements.^32^ In this study, the crystallite quality of the Y doped TiO_2_ nanoparticles with 5 wt% of Y were better than the crystallite quality of the other sample of TiO_2_ with a doping concentration of 1 wt% and 4 wt% of Y. However, the value of the FWHM showed that pure TiO_2_ ≅ Y doped TiO_2_ at the strongest peak of 5 wt% doping, meaning that the FWHM rose slightly when the doping concentration was increased.^17^

**Fig. 12:**
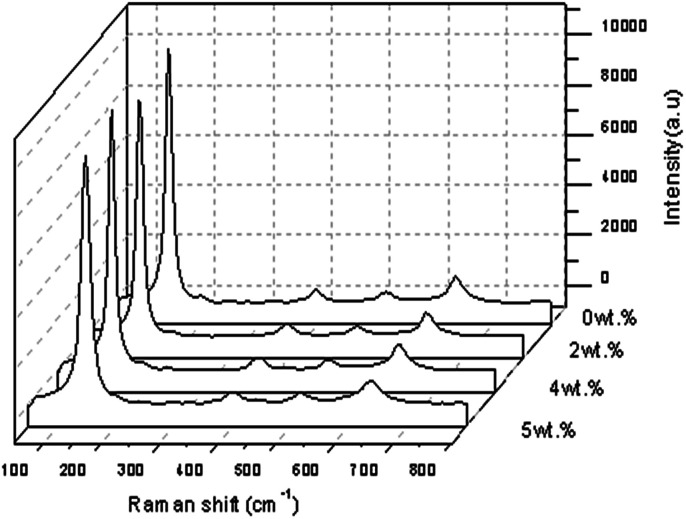
Raman spectroscopy scan for Gd doped TiO_2_ thin film.

Fig. 12 shows the Raman spectra of the TiO_2_ with a Gd doping concentration of 2 wt%, 4 wt% and 5 wt% at a spectral range of 100–800 cm^−1^ with the highest intensity of Raman peaks of anatase TiO_2_ when a 4 wt% Gd doping concentration was applied. Four normal modes were observed at 144.913 cm^−1^ (E_g_), 395.953 cm^−1^ (B_1g_), 514.128 cm^−1^ (a combination of A_1g_ and B_1g_) and 636.698 cm^−1^ (E_g_). The sharpest and strongest peak at about 144.913 cm^−1^ was assigned to the high frequency branch of the E_g_ mode of TiO_2_. This indicated good crystallinity. Thus, from the Raman studies it was determined that the anatase phase was not altered by the presence of a trivalent gadolinium dopant. The change in the position and shape of the Raman active E_g_ line of anatase TiO_2_ was also detected so that the doping concentration was thought to be the main aspect that could produce the lattice distortion of the crystals, which was normally different from the ionic radii of different elements. In this research, the crystallite quality of the Gd doped TiO_2_ with a doping concentration of 5 wt% of Gd was better than the crystallite quality of the other samples of TiO_2_ with 2 wt% and 4 wt% Gd doping concentrations. However, the value of FWHM showed Gd doped TiO_2_ < pure TiO_2_ at the strongest peak of 5 wt%, meaning that FWHM decreased slightly when the doping concentration increased.^33^

XPS was carried out to check the surface chemical composition, purity and the oxidation valence states of TiO_2_, and Al doped TiO_2_. In order to understand the mechanism resulting in the changes in the band gap of Al doped TiO_2_ films, the films were investigated using XPS. The XPS being a surface sensitive technique provided information about the change in the chemical state of the constituent species of film. Here, the variation in the chemical state of the O and Ti elements was determined in detail to correlate it to the observed variations in the band gap of the films.^34^

**Fig. 13:**
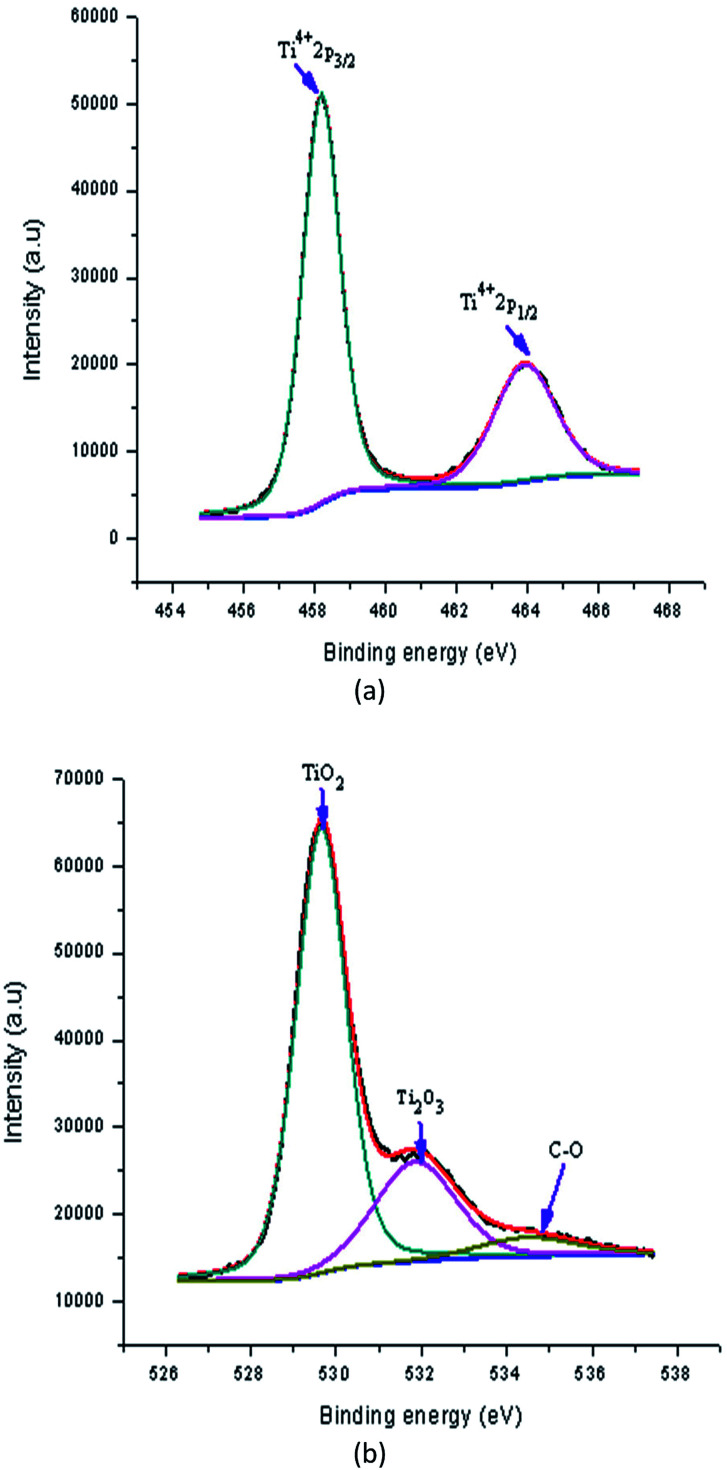
High resolution XPS spectrum of pure TiO_2_ film (a) Ti2p and (b) O1s.

Fig. 13(a) shows a high resolution XPS spectrum of pure TiO_2_ film. In this spectrum, the doublet Ti2p_3/2_ (binding energy 458.20 eV) and Ti2p_1/2_ (binding energy 463.92 eV) occurs from the spin–orbit splitting. These peaks were consistent with Ti^4+^ in the TiO_2_ lattice. Next, the O1s spectrum of pure TiO_2_ thin film is shown in Fig. 13(b), which was fitted the three peaks. The peaks at binding energies of 529.65 eV, 531.85 eV and 534.50 eV were attributed to lattice oxygen, titanium(iii) oxide (Ti_2_O_3_) and non-lattice oxygen, respectively.

**Fig. 14:**
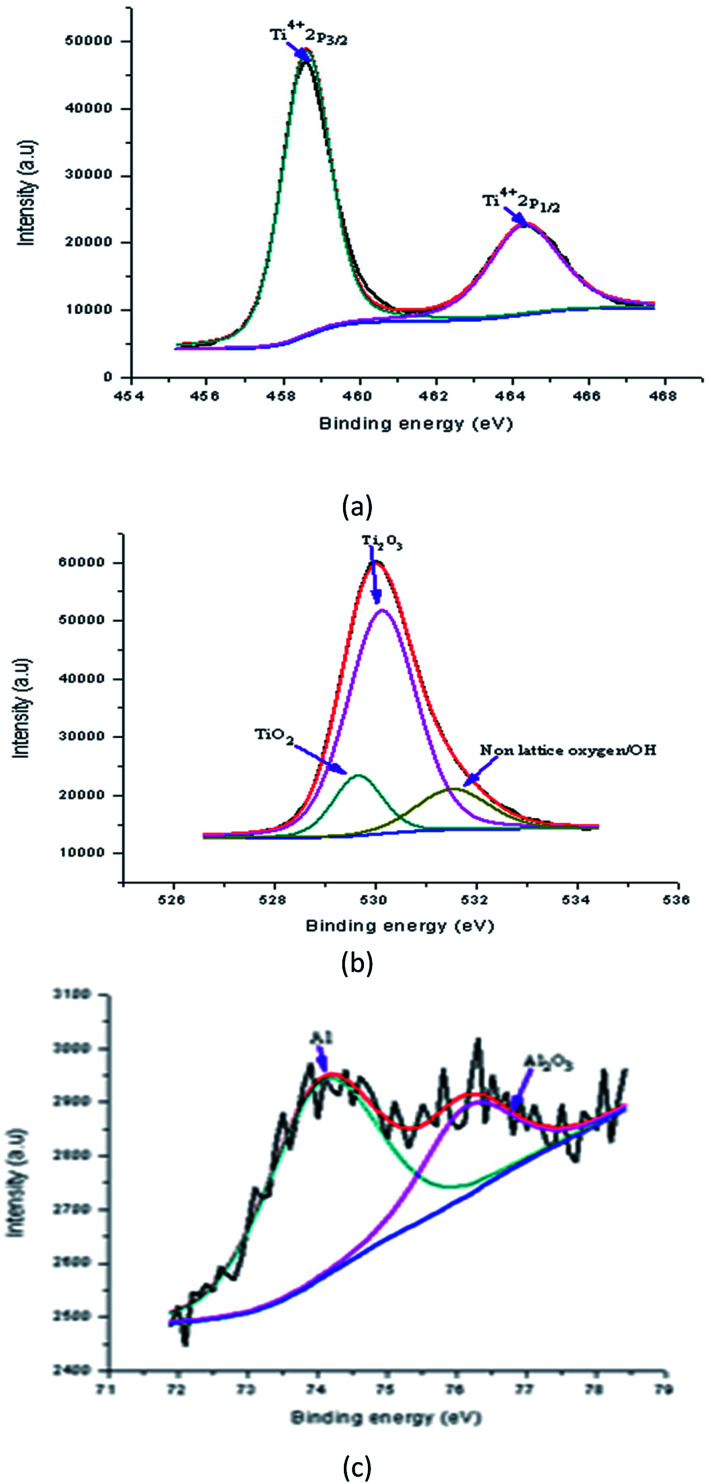
High resolution XPS spectra of Al doped TiO_2_ film (a), Ti2p (b), O1s and (c) Al2p and Al2s.

Fig. 14(a) shows the peaks in the Al doped samples now located at binding energies of 458.60 eV (Ti2p_3/2_) and 464.32 eV (Ti2p_1/2_). The sample was 3 wt% Al doped TiO_2_ thin films. The alteration in the position of these peaks shows the effect of an Al increase on the electronic state of the Ti element. It is most likely that some of the Ti ions are replaced with Al ions in the lattices. The rise in the area of the Ti^4+^ peak shows that either TiO_2_ was created in a large amount or some of the oxide structure mingled with the Al (having oxidation state Ti^4+^) which was created after the doping. Similarly, for the doped sample, the O1s spectrum of Al doped TiO_2_ thin film fitted with three peaks is shown in Fig. 14(b). In this spectrum, three peaks at binding energies of 529.65 eV, 530.13 eV and 531.52 eV were detected which were assigned to lattice oxygen, lattice oxygen (Ti_2_O_3_) and lattice oxygen (Ti_2_O_3_ + OH), respectively. This shows that in the doping process the TiO_2_ is created together with some mixed oxide.

The alteration in stoichiometry was expected because of the change in area of the relative peaks. The Ti^3+^ state existed in the Al doped TiO_2_ in which the oxygen vacancy defects were created. Al doping results in a small alteration in the binding energy, revealing that the Al ions were better dispersed in the replacement sites of the TiO_2_ lattice and more mingled oxide structure, which could be Al–O–Ti. Fig. 14(c) shows the XPS spectrum at binding energies of 74.07 eV and 76.15 eV corresponding to the Al2p and Al2s of Al doped TiO_2_ film, respectively. The appearance of these peaks supports the existence of Al in the Al^3+^ ionic state.^35^

**Fig. 15:**
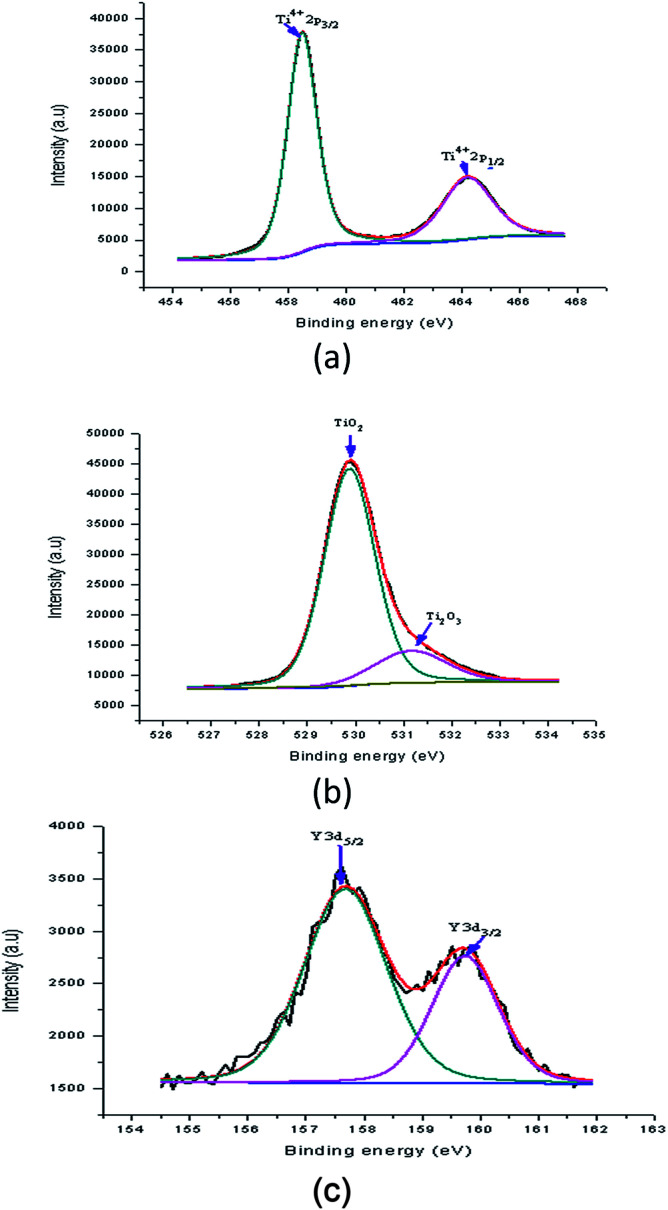
High resolution XPS spectrum of Y doped TiO_2_ film (a) Ti2p, (b) O1s, and (c) Y3d.

Fig. 15(a) shows the peaks in the Y doped samples that were detected at binding energies of 458.49 eV (Ti2p_3/2_) and 464.19 eV (Ti2p_1/2_). These peaks were assigned to 4 wt% Y doped TiO_2_. The shift in the position of these peaks shows the effect of a Y increase on the electronic state of the Ti element. Possibly some of the Ti ions were replaced with Y ions in the lattices. For the Y doped sample, the O1s spectrum of the Y doped TiO_2_ thin film fitted with three peaks is shown in Fig. 15(b). In this spectrum, two peaks at binding energies of 529.88 eV and 531.15 eV were observed which were assigned to lattice oxygen (TiO_2_) and Ti_2_O_3_, respectively. The Ti^3+^ state existed in the Y doped TiO_2_ in which oxygen vacancy defects were created. This showed that in the doping process TiO_2_ was created together with some mingled oxide. The alteration in stoichiometry was expected because of the change in area of the relative peaks. Nevertheless, the peak at a binding energy of 530.03 eV appeared to correspond to the lattice oxygen. The Y doping concentration results in a small alteration in the binding energy, showing that Y ions were better dispersed in the replacement sites of the TiO_2_ lattice and created a more mingled oxide structure, which could be Y–O–Ti. Fig. 15(c) shows the XPS spectrum at binding energies of 157.68 eV and 159.74 eV which corresponded to Y3d_5/2_ and Y3d_3/2_, respectively, of the Y doped TiO_2_ thin film. The creation of these peaks preserved the existence of Y in the Y^3+^ ionic state. These peaks also show that the usual Y3d spectra with spin-doublets (d_3/2_ and d_5/2_) were separated by two peaks, both of 2.06 eV.^36^

**Fig. 16:**
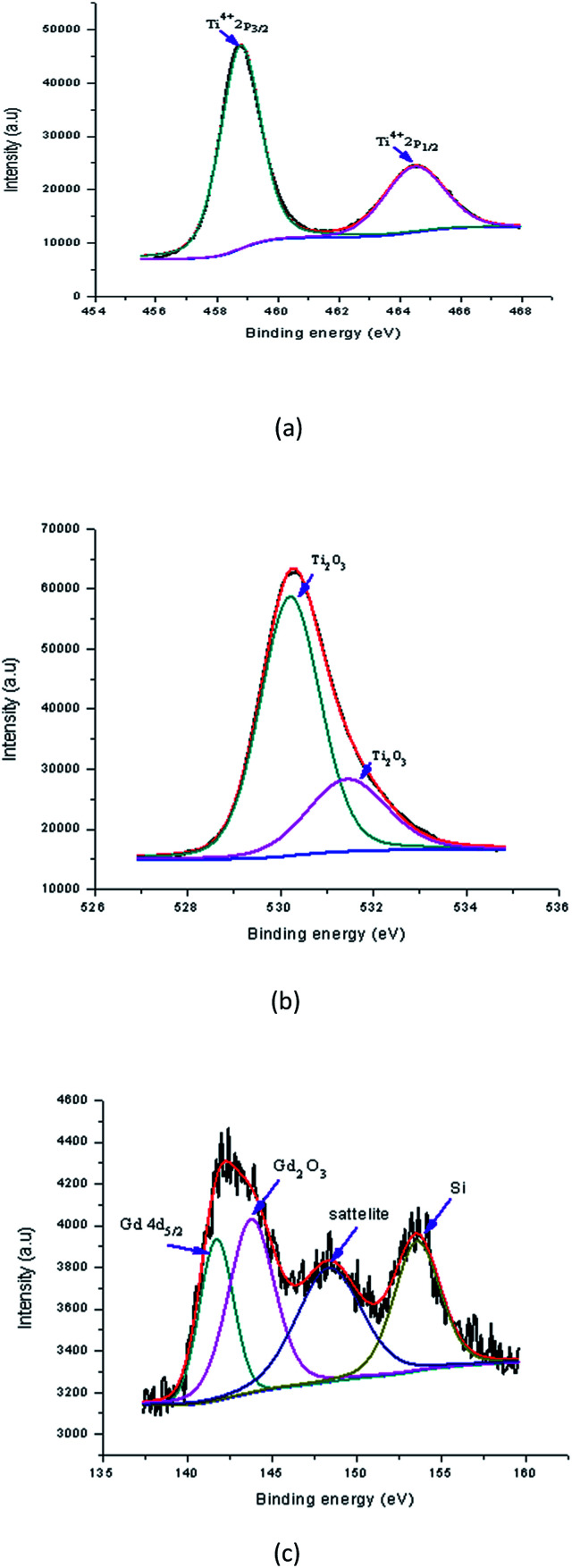
High resolution XPS spectra of Gd doped TiO_2_ film (a) Ti2p, (b) O1s, and (c) Gd4d.

Fig. 16(a) shows the peaks in the Gd doped samples that were found at binding energies of 458.77 eV (Ti2p_3/2_) and 464.49 eV (Ti2p_1/2_). The alteration in the position of these peaks shows the effect of Gd accumulation on the electronic state of the Ti element. Possibly some of the Ti ions were replaced with Gd ions in the lattices. The area of the Ti^4+^ peak shows that either TiO_2_ was created in a large amount or some mingled oxide structure with Gd (having the oxidation state of Ti^4+^) was created after doping. Meanwhile, the reducing area of the Ti^4+^ indicated a reduction of TiO_2_ in the sample, and probably the creation of the Ti–O–Gd structure in the TiO_2_ lattice through the replacement of transition metal ions. The detected alteration in the peaks also showed contact between the Ti and Gd atoms and an overlapping of their 4d orbital. Fig. 16(b) shows the Gd doped sample, in which the O1s spectrum of the Gd doped TiO_2_ thin film was fitted with two peaks that were detected at binding energies of 530.21 eV and 531.43 eV which were assigned to lattice oxygen (Ti_2_O_3_) and lattice oxygen (Ti_2_O_3_), respectively. The Ti^3+^ state existed in the Y doped TiO_2_ in which the oxygen vacancy defects were created. This showed that defects in the doping process of TiO_2_ were created together with some mingled oxide. The alteration in stoichiometry was expected because of the alteration in the area of the relative peaks. Nevertheless, the TiO_2_ peak at the binding energy 529.91 eV appeared to correspond to the lattice energy. Fig. 16(c) revealed that the Gd ions were better dispersed in the replacement sites of the TiO_2_ lattice and created a more mingled oxide structure, which could be Gd–O–Ti. This also showed that the XPS spectrum at binding energies of 141.73 eV and 143.79 eV corresponded to Gd_4_d_5/2_ and gadolinium(iii) oxide (Gd_2_O_3_) of the Gd doped TiO_2_ film, respectively. The creation of these peaks encouraged the existence of Gd in the Gd^3+^ ionic state. A shake up satellite peak at 148 eV also encouraged Gd to be exhibited in the Gd^3+^ state as an oxide. These shake-up satellite peaks were related to the Gd3d–O2p hybridisation. Thus, the XPS analysis indicated that Gd ions were doped into the TiO_2_ matrix in the form of Gd–O–Ti.^37^

## Conclusions

Pure titanium dioxide (TiO_2_) and doped TiO_2_ thin films were prepared by spin coating titanium(iv) butoxide on a glass substrate from a sol–gel, followed by annealing at 500 °C. The anatase phase was observed to be annealed at 500 °C. The doped TiO_2_ thin film was produced by doping with metal atoms for which the ionic state was a 3+ ion, which were Al, Gd, and Y. The ionic state, 3+, of the metal atom doping based on lattice distortion could contribute oxygen vacancy defects that provided advantages to the thin film. The conductivity increase because of a faster growth of the thin film encouraged the formation of a higher level of oxygen vacancy defects. These thin films can be applied in gas sensor applications.

## Conflicts of interest

There are no conflicts of interest to declare.

## Supplementary Material
